# Identification and molecular characterization of missense mutations in orphan G protein–coupled receptor GPR61 occurring in severe obesity

**DOI:** 10.1016/j.molpha.2025.100026

**Published:** 2025-03-04

**Authors:** Choi Har Tsang, Alexander De Rosa, Paweł Kozielewicz

**Affiliations:** 1 Molecular Pharmacology of GPCRs, Department of Physiology and Pharmacology, Karolinska Institutet, Solna, Sweden; 2 School of Engineering Sciences (SCI), KTH Royal Institute of Technology, Stockholm, Sweden

**Keywords:** G protein–coupled receptors, GPR61, Missense mutations, Severe obesity, Bioluminescence resonance energy transfer, Metabolism, Orphan receptors

## Abstract

Severe obesity is a complex chronic metabolic condition with a body mass index over 40 and can be caused, for example, by dysregulated G protein–coupled receptors (GPCRs) signaling. The orphan GPCR GPR61 had been linked to the regulation of metabolism and, here, we identify 34 mutations in the GPR61 gene which are present with much higher frequency in severe obesity samples from the UK10K obesity screen compared to the normal population. Furthermore, the cumulative sum of GPR61 mutations was found to be higher compared to the highly mutated and well-established target, melanocortin 4 receptor. Some GPR61 mutations presented an impact on ligand-independent GPR61-induced cAMP production. Specifically, R236C^5.66^ compromised G_s_ protein activation and altered the pattern of cellular expression. Our data warrant further studies to assess the role of this orphan GPCR in metabolism in greater detail.

**Significance Statement:**

This study identified missense mutations, including previously unknown variants, of the GPR61 gene in severely obese patients. This occurrence was higher than for the well-established obesity target melanocortin 4 receptor. In the in vitro assays, 3 mutations of GPR61, in particular R236C^5.66^, were loss of function because they reduced the constitutive activity of the receptor. The data support the notion that GPR61 can act as a promising target in obesity and its functions should be explored in future studies.

## 1 Introduction

Obesity is a disease defined as “abnormal or excessive fat accumulation that presents a health risk, with a body mass index (BMI) over 30” (World Health Organization), whereas, severe obesity is defined as a BMI >40 (https://www.nhs.uk/conditions/obesity/). Obesity is linked to several metabolic problems, including type 2 diabetes and cardiovascular diseases, which contribute to significant morbidity, mortality, and increased healthcare costs (Heymsfield and Wadden, 2017). Furthermore, it is associated with abnormal regulation of appetite (Roger et al, 2022; Suzuki et al, 2012), and in the human body, the central control of food intake takes place in the hypothalamus (Roger et al, 2022; Suzuki et al, 2012). As such, signaling mediated by hypothalamus-expressed membrane proteins from the G protein–coupled receptors (GPCRs) family has been shown to regulate food intake and metabolism (Adan et al, 2006; Tao, 2014; Lotta et al, 2019; Barella et al, 2021; Deng et al, 2021). The key pathway in appetite regulation is the proopiomelanocortin/melanocortin 4 receptor (MC_4_R) circuit (Adan et al, 2006; Baldini and Phelan, 2019; Brouwers et al, 2021). Ligand-induced and constitutive activity of wild-type (WT) or gain-of-function mutants of MC_4_R lead to the activation of transducer G_s_ proteins and an increase in intracellular cAMP production, which results in reduced food intake (Adan et al, 2006; Tao, 2014; Lotta et al, 2019). Conversely, brain-expressed agouti-related protein acts as an inverse agonist of MC_4_R blocking its signaling (Nijenhuis et al, 2001). Interestingly, nonspecific G_s_ stimulation in agouti-related protein neurons leads to increased food intake. This suggests that understanding the details of GPCR-G_s_ protein signaling can be key in elucidating molecular mechanisms of the development of obesity (Nakajima et al, 2016).

GPR61 belongs to the class A orphan GPCRs, ie, receptors for which endogenous ligands have not been identified and verified by studies from at least 2 independent laboratories. Here, the incentive to investigate GPR61 was that its role in regulation of metabolism has already been proposed but the receptor is far from being established as a functional regulator of metabolism. To this end, the fundamental—in the light of the hypothesis underlying our study—publication from 2011 reported that the GPR61 knockout mice developed significant hyperphagia and an increased body weight in comparison to the WT mice (Nambu et al, 2011). Although, to some surprise, this study has not been followed up by more in-depth mechanistic reports, several publications as well as genome-wide association studies (GWAS) have indeed predominantly associated GPR61 gene locus with type 2 diabetes, increased BMI, and body fat composition (https://www.ebi.ac.uk/gwas/genes/GPR61) (Yuan et al, 2014; Felix et al, 2016). To date, only little is known about GPR61-mediated signaling but it is recognized that overexpressed GPR61 constitutively couples to heterotrimeric G_s_, similarly to MC_4_R (Takeda et al, 2003; Kozielewicz et al, 2019). Next, this orphan GPCR has been shown to form heterodimers with melatonin MT_2_ receptors (Oishi et al, 2017). Stimulation of cells coexpressing these 2 receptors with melatonin leads to reduced *β*-arrestin2 recruitment to MT_2_ and decreased cellular cAMP levels. Moreover, and again analogously to MC_4_R, the N-terminus of GPR61 plays an important role in its constitutive activity (Toyooka et al, 2009; Ersoy et al, 2012; Sveidahl Johansen et al, 2021). Furthermore, the receptor is subject to *N*-glycosylation at one asparagine (N12) position (Kozielewicz et al, 2017). More recently, the structures of GPR61 were resolved by cryogenic electron microscopy using a receptor lacking the N-terminus, the intracellular loop 3 (ICL3), and the C-terminus (Lees et al, 2023; Nie et al, 2023). In these studies, the constitutive activity of the receptor was attributed to the extracellular loop 2 penetrating into the putative orthosteric binding pocket. Lastly, one weak and one potent synthetic inverse agonist have been introduced but the pharmacological toolbox for GPR61 is still very limited (Takeda et al, 2003; Kozielewicz et al, 2019; Lees et al, 2023).

In our study, we analyzed 480 samples from UK10K obesity datasets containing sequencing information from severe obesity cases (https://www.uk10k.org/studies/obesity.html) and we found 34 missense mutations in the GPR61 gene. Some of these mutations are not present and the rest occur with much higher frequency in diseased individuals than in the normal population (Genome Aggregation Database [gnomAD]). These mutations had an impact on the constitutive activity of GPR61. Specifically, the R236C^5.66^ mutation led to a significant decrease in the constitutive activity of GPR61 in Förster/fluorescence resonance energy transfer (FRET)–based cAMP, enhanced bystander bioluminescence resonance energy transfer (ebBRET)–based G_s_ translocation assays, and altered receptor’s cellular expression profile Furthermore, molecular dynamics (MD) simulations revealed distinct conformational changes in the transmembrane domain structure of the mutated receptor in comparison with the WT counterpart.

In conclusion, this study increases the knowledge about GPR61 and contributes to the growing body of data that this orphan receptor presents another G_s_-coupled receptor with a potential role in regulating metabolism. As such, future studies should address the role of this receptor and its mutations in more detail, and in relevant models.

## 2 Materials and methods

### 2.1 Common variant association with BMI and weight

We utilized the Common Metabolic Diseases Knowledge Portal (CMDKP; https://www.hugeamp.org) and downloaded lists of 1000 genes which common variants are significantly associated with the following: (1) BMI (https://hugeamp.org/phenotype.html?phenotype=BMI); *P* ≤ 2.57 × 10^−9^; and (2) weight (https://hugeamp.org/phenotype.html?phenotype=WEIGHT) *P* ≤ 1.95 × 10^−10^. The common variant gene-level associations are calculated from bottom-line common variant genetic associations using the MAGMA algorithm. Nine GWAS or ExSeq studies have been meta-analyzed for weight and 70 such studies have been meta-analyzed for BMI. The sample size for GPR61 in the BMI association calculations was 2,249,902 and in the weight association calculations was 396,218. A generally accepted threshold for the significance of MAGMA results is *P* ≤ 2.5 × 10^−6^.

Next, we used the list of all of the GPCR targets (https://www.guidetopharmacology.org/DATA/GPCRTargets.csv) and screened the CMDKP data for all GPCRs gene names as listed by the HUGO Gene Nomenclature Committee.

### 2.2 UK10K data base search and analysis

We leveraged the UK10K dataset, accessed via the European Genome-Phenome Archive (EGA; dataset IDs: EGAD00001000429, EGAD00001000431, EGAD00001000432; application agreement 15267), to perform comprehensive mutational analysis and single nucleotide polymorphism (SNP) calling. This dataset was selected for its broad genomic coverage, generated using the Illumina HiSeq 2000 platform, and its relevance to the cohort under study, which included individuals with a BMI ≥40 or participants in the Severe Childhood Onset Obesity Project. A total of 480 samples (308 from The Severe Childhood Onset Obesity Project, 64 from the TwinsUK study, and 108 from The Generation Scotland: Scottish Family Health Study) were available for download and subsequent processing. To ensure data security and integrity, all samples were securely downloaded using the EGA Live Outbox tool. Following this, a series of stringent preprocessing steps were applied to each data sample. The processed reads were then aligned to the GRCh38 human reference genome using TopHat, ensuring high-quality mapping (Kim et al, 2013). Acceptable hits were subsequently processed using Samtools mpileup, which provided the foundation for accurate variant detection. For SNP calling and variant detection, we utilized VarScan mpileup, with downstream results converted into interpretable formats using SNPsift (Koboldt et al, 2012). This analytical pipeline ensured precise identification of genetic variants, providing a reliable framework for subsequent analyses. Identified mutations were then cross-referenced with the National Center for Biotechnology Information Short Genetic Variations database (dbSNP) for validation. Additionally, the Ensembl Variant Effect Predictor was employed to predict the functional consequences of each mutation (McLaren et al, 2016). This tool facilitated the assessment of the potential impact on protein-coding regions, regulatory elements, and associations with known phenotypes, allowing for an in-depth understanding of the genetic variations in this cohort. As controls representing the normal population, we used variants from over 730,000 individuals from gnomAD v4.1.0 (www.gnomad.broadinstitute.org).

### 2.3 In vitro cell culture

Human embryonic kidney 293A (HEK293A) cells (Thermo Fisher Scientific) were cultured in Dulbecco’s modified Eagle’s medium supplemented with 10% FBS (Sigma), 1% penicillin/streptomycin, 1% L-glutamine (both from Thermo Fisher Scientific) in a humidified CO_2_ incubator at 37 °C. All cell culture plastics were from Sarstedt, unless otherwise specified. Plates were not coated prior to seeding cells. The absence of mycoplasma contamination was routinely confirmed by polymerase chain reaction using 5′-GGCGAATGGGTGAGTAACACG-3′ and 5′-CGGATAACGCTTGCGACTATG-3′ primers detecting 16S ribosomal RNA of mycoplasma in the media after 2–3 days of cell exposure.

### 2.4 DNA constructs, cloning, and mutagenesis

HiBiT-GPR61 plasmid DNA was generated with Gibson cloning using a codon-optimized GPR61 from GPR61-Tango plasmid DNA (#66366 Addgene, deposited by Bryan Roth) as an insert and HiBiT-FZD_6_ with a 5-HT_3_A signal peptide plasmid DNA as a backbone (Kozielewicz et al, 2021). In the newly generated construct, the GPR61 insert sequence replaced the FZD_6_ insert sequence. In this construct, the N-terminally cloned HiBiT tag (GTGAGCGGCTGGCGGCTGTTCAAGAAGATTAGC) is followed by a GS linker (GGATCC, BamHI site). HiBiT-GPR61-nanoluciferase (Nluc) was generated using Gibson cloning, inserting Nluc from Nluc-FZD_6_ (Kozielewicz et al, 2020) onto the C-terminus of HiBiT-GPR61, without a linker. Plasmid DNA constructs encoding different receptor mutants were generated with a GeneArt Site-Directed Mutagenesis kit (Thermo Fisher Scientific). rGFP-CAAX plasmid DNA, rGFP-FYVE plasmid DNA, rGFP-giantin plasmid DNA, rGFP-PTP1B plasmid DNA, Rap1Gap-*R*luc2 and G*α*s-67-*R*luc2 plasmid DNA in the pcDNA3.1(+) backbone were synthesized by GenScript. Plasmid DNA encoding an *α* subunit of G_i1_ was from cDNA.org. Salmon sperm DNA (ss DNA) was from Thermo Fisher Scientific. Exchange protein activated by cAMP (EPAC)–based cAMP sensor H187 plasmid DNA (Klarenbeek et al, 2015) was a kind gift from Kees Jalink (The Netherlands Cancer Institute). Venus-KRas plasmid DNA, Venus-Rab5 plasmid DNA, Venus-Rab11 plasmid DNA, Venus-giantin plasmid DNA, and Venus-PTP1B plasmid DNA were kind gifts from Nevin Lambert (Augusta University). The constructs were validated by Sanger sequencing (Eurofins GATC).

### 2.5 Total and cell surface expression of HiBiT-GPR61 constructs

HEK293A cells were transiently transfected in suspension using polyethylenimine (PEI, Polysciences). A total of ca. 4 × 10^5^ cells were transfected in 1 mL with 200 ng of HiBiT-GPR61 WT or mutants plasmid DNA and 80 ng of ss DNA, or only 280 ng of ss DNA. Next, transfected cells (4 × 10^4^ cells in 100 *μ*L) were seeded onto white 96-well cell culture plates. Twenty-four hours later, the cells were washed once with 200 *μ*L of Hanks’ balanced salt solution (HBSS) (HyClone). Next, 50 *μ*L of HBSS was added to the wells. Subsequently, to measure the total cellular expression of HiBiT-tagged receptors, 50 *μ*L of HiBiT lytic detection system buffer (Promega) with 1:50 dilution of furimazine and 1:100 dilution of LgBiT (Promega) were added. To measure cell surface expression of HiBiT-tagged receptors, 50 *μ*L of HiBiT extracellular detection buffer (Promega) with 1:50 dilution of furimazine and 1:100 dilution of LgBiT were added to adjacent wells transfected with the same plasmid DNA mix. The plate was incubated for 15 minutes at room temperature on an orbital shaker at 150 rpm, and subsequently, the NanoBiT emission (460–500 nm, 200 ms integration time) was measured in 3 consecutive reads using a Spark microplate reader (TECAN) at room temperature.

### 2.6 FRET-based cAMP production

HEK293A cells were transiently transfected in suspension using Lipofectamine 2000 (Thermo Fisher Scientific). A total of ca. 4 × 10^5^ cells were transfected in 1 mL with 200 ng of HiBiT-GPR61 constructs, 100 ng EPAC-based cAMP sensor, and 700 ng of pcDNA3.1 plasmid DNA. Next, transfected cells (4 × 10^4^ cells in 100 *μ*L) were seeded onto black 96-well cell culture plates. Twenty-four hours later, the cells were washed once with 200 *μ*L of HBSS (HyClone). Next, 100 *μ*L of HBSS was added to the wells, and the FRET ratio was measured in 3 consecutive reads with a CLARIOstar plate reader (BMG) at room temperature. mTurquoise2 (donor) was excited at 410 to 450 nm and its emission intensity using a 460–500 nm monochromator. cpVenus173 (acceptor) emission was recorded using a 515–555 nm monochromator. FRET ratios were defined as acceptor emission/donor emission.

### 2.7 G protein activation with ebBRET

HEK293A cells were transiently transfected in suspension using PEI. To measure G_s_ protein activation, a total of ca. 4 × 10^5^ cells were transfected in 1 mL with 50 ng (low overexpression), 200 ng (standard overexpression) or 500 ng (high overexpression) of HiBiT-GPR61 plasmid DNA constructs, 300 ng of rGFP-CAAX/rGFP-FYVE/rGFP-giantin/rGFP-PTP1B plasmid DNA, 40 ng of G*α*s-67-Rluc2 plasmid DNA and 610 ng, 460 ng, or 160 ng of pcDNA3.1 plasmid DNA. To measure, G_i_ protein activation, a total of ca. 4 × 10^5^ cells were transfected in 1 mL with 200 ng of HiBiT-GPR61 DNA constructs, 300 ng of rGFP-CAAX plasmid DNA, 100 ng of an *α* subunit of G_i1_ plasmid DNA and 40 ng of Rap1Gap-*R*luc2 plasmid DNA. Next, transfected cells (4 × 10^4^ cells in 100 *μ*L) were seeded onto white 96-well cell culture plates. Twenty-four hours later, the cells were washed once with 200 *μ*L of HBSS (HyClone). Next, 90 *μ*L of HBSS was added to the wells and subsequently, 10 *μ*L of coelenterazine 400a (2.5 *μ*M final concentration, Biosynth) was added. The plate was incubated for 10 minutes. Next, *R*luc2 emission (donor, 360–440 nm, 100 ms integration time) and rGFP emission (acceptor, 505–575 nm, 100 ms integration time) were measured in 3 consecutive reads using Spark microplate reader (TECAN) at room temperature. The bioluminescence resonance energy transfer (BRET) 2 ratios were defined as acceptor emission/donor emission.

### 2.8 Subcellular expression of HiBiT-GPR61-Nluc WT and R236C^5.66^ with bystander BRET

HEK293A cells were transiently transfected in suspension using PEI. A total of ca. 4 × 10^5^ cells were transfected in 1 mL with 100 ng of HiBiT-GPR61-Nluc WT or R236C^5.66^ DNA constructs, 300 ng of Venus-KRas/Venus-Rab5/Venus-Rab11/Venus-giantin/Venus-PTP1B plasmid DNA and 600 ng of pcDNA3.1 plasmid DNA. Next, transfected cells (4 × 10^4^ cells in 100 *μ*L) were seeded onto white 96-well cell culture plates. Twenty-four hours later, the cells were washed once with 200 *μ*L of HBSS (HyClone). Next, 90 *μ*L of HBSS was added to the wells and subsequently, 10 *μ*L of furimazine (1:1000 final dilution, Promega) was added. The plate was incubated for 10 minutes. Next, Nluc emission (donor, 460–500 nm, 200 ms integration time) and Venus emission (acceptor, 520–560 nm, 200 ms integration time) were measured in 3 consecutive reads using the Spark microplate reader (TECAN) at room temperature. The BRET ratios were defined as acceptor emission/donor emission. delta BRET (ΔBRET) ratios were defined as ([(BRET ratio_Nluc- and Venus-cotransfected cells_) − (BRET ratio_Nluc-transfected cells_)]/[BRET ratio_Nluc-transfected cells_]).

### 2.9 MD simulations

MD simulations of GPR61 were conducted using a high-resolution structural model (Protein Data Bank ID: 8KGK) as input. The simulations were initiated on the CHARMM-GUI server. The unresolved C-terminal region of GPR61, missing from the cryogenic electron microscopy structure, was not modeled and was deliberately excluded from the simulations. To mimic the native membrane environment, a hexagonal phospholipid bilayer was carefully constructed around the protein using a lipid composition representative of a physiological membrane. This bilayer was embedded in a solution of water molecules and 0.15 M NaCl to replicate typical ionic conditions in biological systems. The CHARMM36m force field was employed to model the molecular interactions, ensuring that all protein-lipid and protein-solvent interactions were treated with high accuracy. The protonation states of the residues were assigned at a physiological pH of 7.4, optimizing the protein's charge states for biological relevance. Energy minimization was performed on the system using the steepest descent algorithm to eliminate any steric clashes and relieve strain, followed by a multistep equilibration process. Furthermore, during the production phase, the simulations were conducted under isothermal-isobaric ensemble conditions, ensuring constant pressure and temperature throughout. The system's temperature was regulated at 310 K using the Nose–Hoover thermostat. Each mutational variant and WT of GPR61 underwent triplicate simulations, with each trajectory spanning 200 ns, leading to a total simulation time of 600 ns per variant. Upon completion, all MD trajectories were analyzed using Chimera (version 1.17.3) or Visual Molecular Dynamics. Root mean square deviations from individual runs can be found in Supplemental Figs. 1–3. PDB files with final conformations are attached to this publication as Supplemental Material: GPR61 WT simulations: WT_1.pdb, WT_2.pdb, WT_3.pdb; GPR61 T92P^2.56^ simulations: T92P_MD1.pdb, T92P_MD2.pdb, T92P_MD3.pdb; GPR61 R236C^5.66^ simulations: R236C_MD1.pdb, R236C_MD2.pdb, R236C_MD3.pdb; and GPR61 R262C simulations: R262C_MD1.pdb. R262C_MD2.pdb, R262C_MD3.pdb.

### 2.10 Statistical analysis

All data presented in this study come from at least 3 individual experiments (biological replicates) with each individual experiment performed typically at least in duplicates (technical replicates) for each condition, unless otherwise specified in a figure legend. One biological replicate refers to wells containing cells seeded from the same individual cell culture flasks and measured on the same day. Different biological replicates were transfected using separate transfection mixtures. Technical replicates are defined as individual wells with cells from the same biological replicate. Samples were not randomized or blinded during the experiments. Statistical and graphical analyses were performed using GraphPad Prism software (GraphPad). Two datasets were analyzed for statistical differences with an unpaired *t*-test. Three or more datasets were analyzed by one-way ANOVA with Dunnett’s post hoc analysis. Significance levels are given and displayed in the figures as follows: ∗*P* < .05; ∗∗*P* < .01; ∗∗∗*P* < .001; ∗∗∗∗*P* < .0001. Differences between datasets that did not reach statistical significance are left unmarked (ie, there is no “ns”). Data points throughout the manuscript are indicated as the mean ± SD.

## 3 Results

### 3.1 The cumulative sum of GPR61 mutations is higher than for MC_4_R in the analyzed severe obesity samples

**Figure 1:**
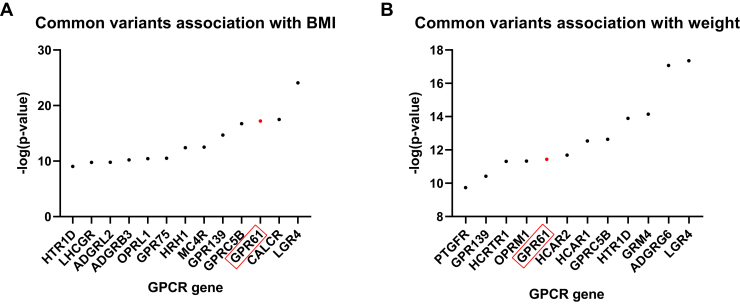
GPR61 gene locus is associated with (A) BMI and (B) weight at genome-wide level of significance. The plots show all the GPCR genes found in the top 1000 genes for each of the 2 associations as analyzed with the CMDKP. Please see the Materials and methods section for more details.

**Figure 2:**
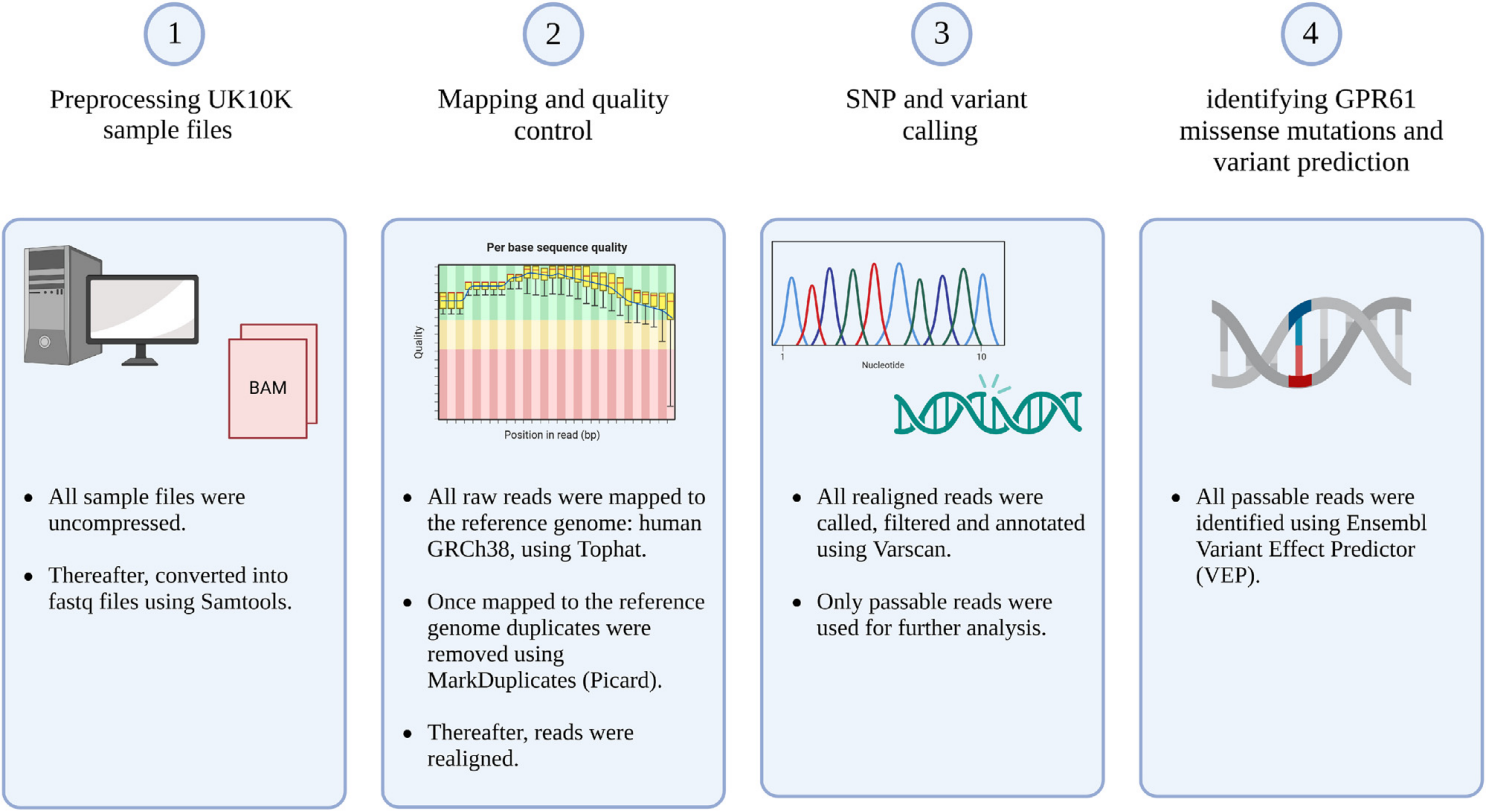
Protocol for exon sequencing and SNP variant calling. The same procedure was used for GPR61 and MC_4_R.

**Figure 3:**
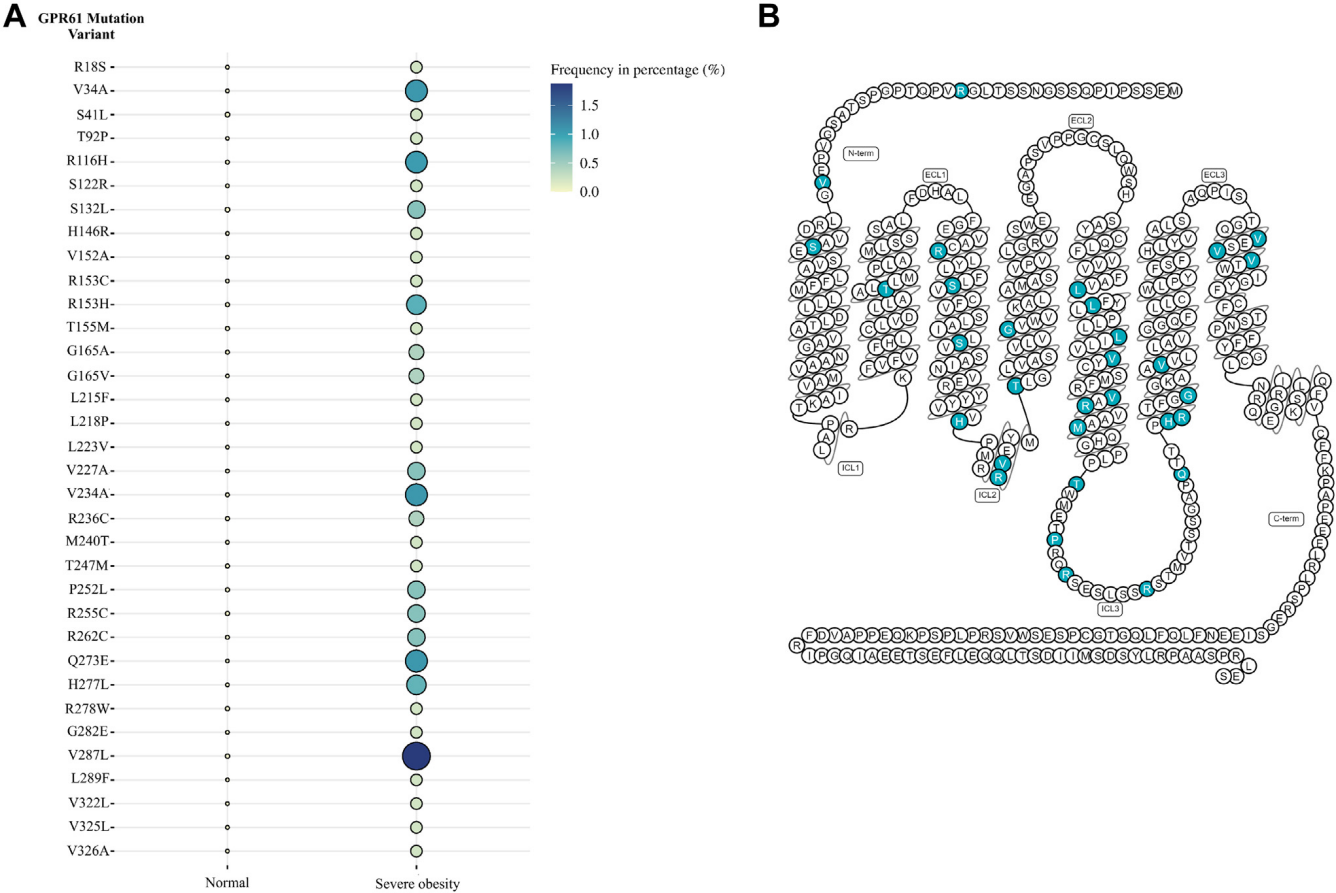
UK10K obesity screen reveals presence of GPR61 mutations. (A) Mutational landscape of GPR61 in normal population and then the same mutations in severe obesity patients. The data are presented as frequencies. The allele counts can be found in Supplemental Fig. 6. (B) GPR61 mutations mapped onto a 2-dimensional model of GPR61.

The analysis of GWAS data with the CMDKP portal revealed that the common variant of the GPR61 gene locus is associated at a genome-wide significance level with BMI and weight, and it is ranked as the top third and the top eighth gene coding for a GPCR (out of 403 analyzed) according to the *P* value, respectively (Fig. 1). These data confirm that the GPR61 gene locus is likely linked to the regulation of metabolism and weight. Because we postulated that GPR61-mediated cellular signaling can play a role in these processes, we hypothesized that disease-linked missense mutations of GPR61, which can be analyzed in functional in vitro assays, can reveal differences in receptor-mediated cellular signaling. These data would fill the knowledge gap and provide new information about processes in which GPR61 is involved. In the next step, we turned our attention to extreme cases of obesity and focused on samples coming from severely obese individuals. From our comprehensive analysis of 480 severe obesity samples obtained from the UK10K study (Fig. 2), we identified 34 different missense mutations in the GPR61 gene (Fig. 3A). Multiple mutations appeared more than once, with the V287L^6.35^ being the mutation with the highest frequency/allele count: in 1.9% of/9 samples. The cumulative sum of GPR61 mutations was 73. Two mutations, R18S and V34A, are found in the N-terminus, a region previously shown to be relevant for this receptor’s activity (Toyooka et al, 2009). However, in the original publication depicting the role of the N-terminus, the R18A mutant did not affect [^35^S]GTPgS binding compared to the WT. The other N-tail mutation we have found, V34A, has not been assessed in that study. Importantly, the following 9 mutations—T92P^2.56^, H146R^3.56^, V152A, G165V^4.40^, L215F^5.45^, L218P^5.48^, L223V^5.53^, Q273E, and L289F^6.37^—were absent from the gnomAD database. Additionally, mutations were localized throughout the polypeptide chain excluding the C-tail (Fig. 3B). Except for S122^3.32^, the amino acid at these positions are not predicted to be involved in the stabilization of the inactive or active state of GPCRs (https://gpcrdb.org/structure_comparison/comparative_analysis#) (Hauser et al, 2021). However, many identified mutations in GPR61 are predicted to have a negative impact on the structure and function of the receptor (Supplemental Fig. 4) (Adzhubei et al, 2013). Next, we extended our analysis also to MC_4_R. As mentioned, MC_4_R is implicated in metabolic regulation and presents a very high mutational rate in obese samples (Vaisse et al, 2000; Doulla et al, 2014; Metzger et al, 2024). Here, we identified 50 missense mutation variants of MC_4_R in the cohort (Supplemental Fig. 5), including previously undescribed mutations. The MC_4_R mutations occurred only one time each. Mutations in GPR61 and MC_4_R displayed as allele counts can be found in Supplemental Fig. 6.

### 3.2 GPR61 mutations have no effect on cellular expression

**Figure 4:**
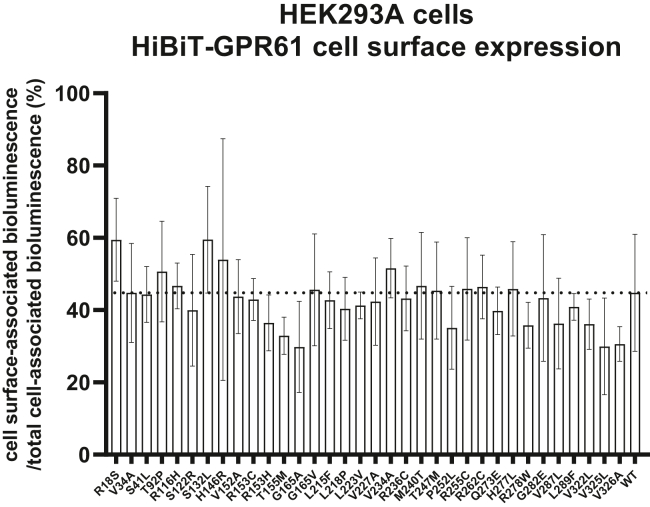
Severe obesity–associated mutations of GPR61 have no statistically significant impact on cell surface trafficking of the overexpressed receptor. NanoBiT-based measurement of total cell and cell surface expression of 34 mutants of GPR61. Data were analyzed for differences between the mutants with the WT by one-way ANOVA with Dunnett’s post hoc analysis. Data are presented as mean of n = 3 independent experiments ± SD. Significance levels are given as: ∗*P* < .05; ∗∗*P* < .01; ∗∗∗*P* < .001; ∗∗∗∗*P* < .0001.

We have generated 34 different HiBiT-GPR61 plasmid DNA constructs, each carrying a single mutation as found in the UK10K obesity datasets analysis. Thereafter, we assessed the impact of the mutations on total cellular expression and cell membrane expression using heterologously overexpressed receptor plasmids in HEK293A cells. The receptor constructs used in this study were N-terminally tagged with HiBiT, which enabled selective quantification of cell surface expression following the addition of cell-impermeable LgBiT and subsequent measurements of cell surface–specific NanoBiT-emitted bioluminescence. Upon cell lysis, the NanoBiT-emitted bioluminescence is a result of the presence of both cell surface and intracellularly located receptors. Using NanoBiT bioluminescence measurements, we analyzed total cell and cell surface expression of the constructs upon overexpression in HEK293A cells (Supplemental Fig. 7). We could demonstrate that, although there was perhaps a tendency for some, none of the 34 mutations reduced neither the total nor the cell surface expression of the overexpressed construct to a degree that reached statistical significance. Similarly, there was no statistically significant difference in an effect on cell surface trafficking between the overexpressed mutants and the overexpressed WT GPR61 calculated as a ratio between bioluminescence emitted from live and permeabilized cells (Fig. 4).

### 3.3 GPR61 R236C^5.66^ reduces the constitutive activity of the receptor

**Figure 5:**
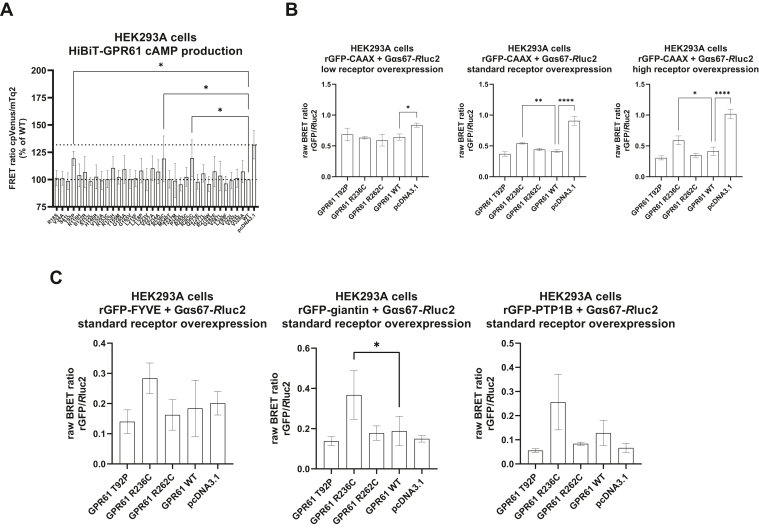
Severe obesity–associated mutations of GPR61 have an impact on cAMP production and G_s_ translocation. (A) EPAC-derived FRET-based biosensor reveals that 3 mutations lead to the statistically significant reduction in cAMP production elicited by overexpression of GPR61 in the absence of an agonist (increase in the FRET ratio reflects decrease in cAMP levels). Data are presented as the mean of n = 4 independent experiments ± SD. (B) The presence of overexpressed GPR61 R236C^5.66^ leads to a statistically significant difference in the bystander BRET ratio between G*α*s-67-*R*luc2 and rGFP-CAAX in comparison with the WT. The reduction in ebBRET signal vs pcDNA3.1 is indicative of G_s_ translocation away (activation) from the cell membrane. The receptor plasmids were overexpressed at 3 different levels to account for any differences in their expression and to confirm that higher receptor expression leads to an increase in its constitutive activity. Data are presented as mean of n = 3 independent experiments ± SD. (C) The presence of overexpressed GPR61 R236C^5.66^ led to statistically significant differences in the bystander BRET ratio between G*α*s-67-*R*luc2 and rGFP-giantin in comparison with the WT. Data were analyzed for differences between the mutants with the WT by one-way ANOVA with Dunnett’s post hoc analysis. Data are presented as mean of n = 3 independent experiments ± SD. Significance levels are given as: ∗*P* < .05; ∗∗*P* < .01; ∗∗∗*P* < .001; ∗∗∗∗*P* < .0001.

Using a genetically encoded, EPAC-derived FRET-based cAMP biosensor, we could show that 3 mutations, T92P^2.56^, R236C^5.66^, and R262C, increased the FRET ratio of the probe in comparison to the WT receptor suggesting inhibition of constitutive GPR61-induced production of cAMP for the N-terminally HiBiT-tagged constructs (Fig. 5A). Therefore, we focused on these 3 mutations and performed an orthogonal ebBRET-based G_s_ translocation assay as a measure of heterotrimeric G_s_ activation (Avet et al, 2022). In these assays, we transfected various amounts of plasmid DNA and showed that these results mirrored the data from cAMP production experiments only for the GPR61 R236C^5.66^ overexpressed at standard and high levels (Fig. 5B). In a set of complementary experiments, we analyzed compartment-specific G_s_ activation and our results revealed that, in addition to the cell membrane, the overexpression of the R236C^5.66^ mutation led to statistically significant differences in the G_s_ activation for receptors located in the Golgi (marked with rGFP-giantin; Fig. 5C). We have already shown that overexpressed HiBiT-GPR61 constructs are present at similar levels at the cell surface. However, we used different, C-terminally Nluc-tagged WT and R236C^5.66^ constructs to assess their subcellular expression. For these HiBiT-GPR61-Nluc WT and R236C^5.66^ constructs, we detected significant differences in receptor expression at the cell membrane which can be explained by higher internalization of the mutant (higher BRET in the Venus-Rab5 setup), and by trapping of the incompletely folded mutant in the endoplasmic reticulum (marked with Venus-PTP1B) as well as the differences in recycling processes as indicated by the higher expression of the WT in the recycling endosomes (marked with Venus-Rab11) (Supplemental Fig. 8). Finally, we analyzed the cell membrane-associated G_i_ activation which revealed that the R236C^5.66^ mutant inhibited G_i_ activation by endogenous HEK293 cell-expressed receptors to a lesser degree than the other 2 mutations and the WT (Supplemental Fig. 9).

### 3.4 MD simulations reveal changes in receptor structure for GPR61 R236C^5.66^

**Figure 6:**
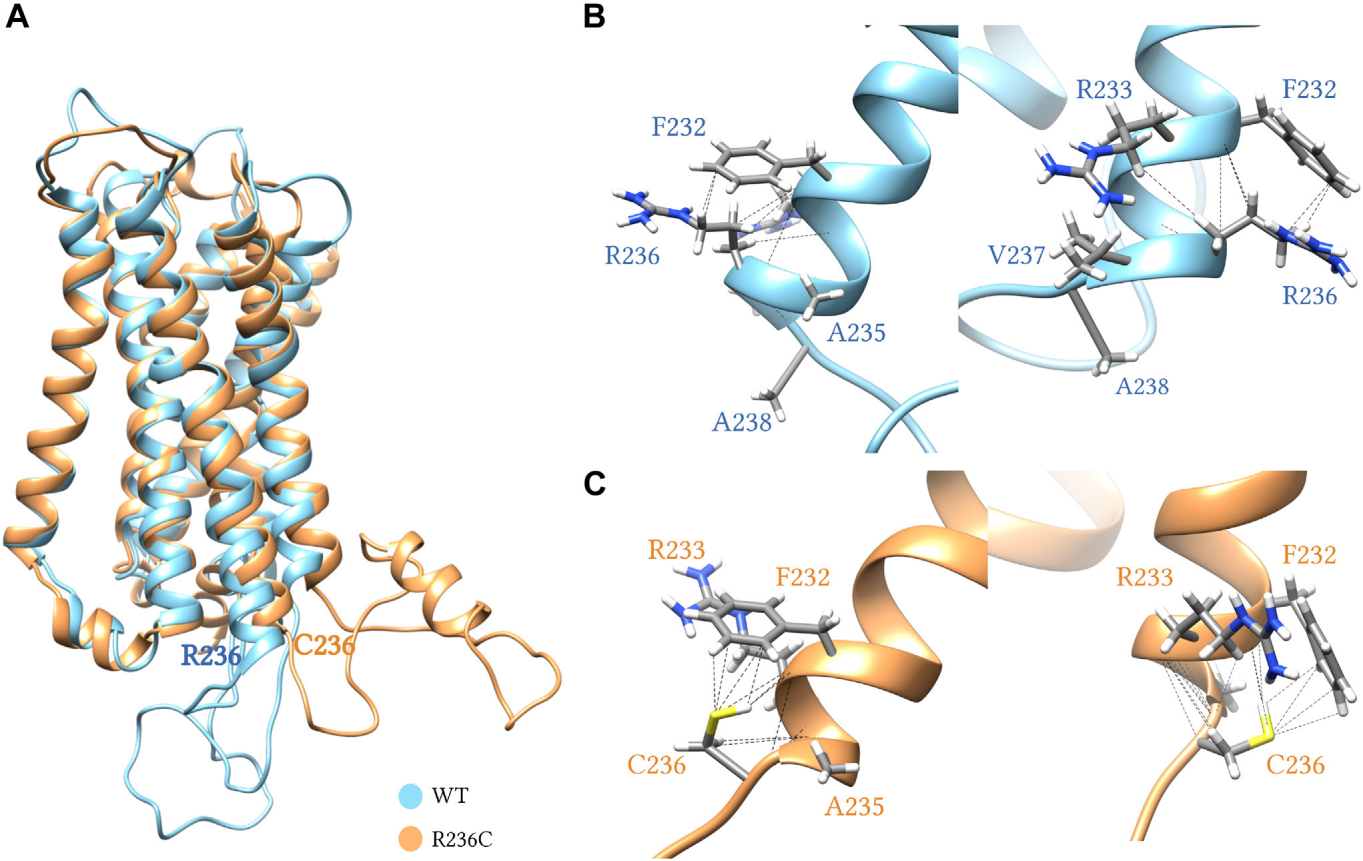
MD simulations predict changes in the structure of the helix 5 following the introduction of the R236C^5.66^ mutation. (A) Final receptor conformations after 200 ns simulation from one representative MD simulation. (B) Zoomed-in region of R236^5.66^ (WT) and (C) C236^5.66^ (mutant) with polar interactions depicted as dashed lines. Root mean square deviations from the independent runs can be found in Supplemental Fig. 1.

In our MD simulations of the R236C^5.66^ mutation, chosen based on the in vitro data from the constitutive activity studies, we observed visible structural deviations compared to the WT protein. Structurally, the GPR61 WT at position 236 has an arginine that presents itself with a helix. The mutant contains a cysteine residue which resulted in a loss of *α*-helical structure, and the presence of different intrahelical interactions (Fig. 6). To this end, in the WT, R236^5.66^ forms Van der Waals, hydrophobic, and polar interactions with F232, polar interactions with R233, polar interactions with A235^5.65^, polar interactions with V237^5.67^ and polar interactions with A238^5.68^. In the mutant, C236^5.66^ forms polar and Van der Waals interactions with F232^5.62^, and polar interactions with R233^5.63^ and A235^5.65^. The MD analysis of the other 2 selected mutants, T92P^2.56^ and R262C, revealed no clear differences (Supplemental Figs. 2 and 3). It has to be noted that the ICL3 has not been resolved in the 8KGK structure and had to be modeled in our study.

## 4 Discussion

The hypothesis of this study was that GPR61 is important for the regulation of metabolism and this role would be reflected by a relatively high number of mutations presented in the samples of severely obese individuals. This hypothesis was tested also by drawing analogies between GPR61 and MC_4_R. MC_4_R is a well-characterized receptor involved in regulating appetite and its variants are the key contributors to monogenic obesity, affecting satiety signals and homeostasis energy expenditure (Farooqi et al, 2003; Doulla et al, 2014; Lotta et al, 2019; Aykut et al, 2020; Brouwers et al, 2021; Metzger et al, 2024). Another incentive for this study was to test the notion emerging from a few previously published studies that GPR61 has a role in metabolic regulation and if so, postulate that it can be grouped with other GPCRs with established links to obesity (eg. glucagon-like peptide-1 receptor). The search for novel targets to treat metabolic diseases is ongoing as shown by a very recent study which identified neurokinin 2 receptor, a G_s_- and G_q_-coupled receptor, as another potentially relevant protein for the regulation of body weight and blood glucose (Sass et al, 2024). Our study was not aimed to assess or to prove causation between the presence of GPR61 mutations and (severe) obesity. In general, it has been shown that obese individuals have an increased rate of DNA instability, elevated number of mutations, and dysregulation in the cAMP/PKA signaling axis (London and Stratakis, 2022; Loos and Yeo, 2022; Kompella et al, 2024).

First, we showed that GPR61 gene locus is linked with regulation of metabolism by reporting genome-wide significance of its association with BMI and weight. Then we analyzed samples from the UK10K obesity screen for the presence of missense mutations in the GPR61 gene and compared them with the normal population found in the gnomAD database. We compared the frequency of mutations in GPR61 with MC_4_R to assess whether similar patterns of mutation frequency could be observed in the gene with the established role in obesity and metabolic disorders. The cumulative allele frequency is comparable to MC_4_R, which has a well-documented role in obesity (Sawabe et al, 2024). We use this analogy to suggest that the high frequency of mutations observed in GPR61, and even the higher cumulative number of mutations than for MC_4_R, may indicate the role of GPR61 in metabolism/obesity. In fact, there were 16 mutations in GPR61 which occurred more than once in our analysis, whereas each of the identified MC_4_R mutations appeared only once. Moreover, the absence of some of the mutations in gnomAD underlines that they have not yet been documented in the broader genetic landscape. Further studies should explore the receptor’s functions in these pathophysiological conditions.

Thereafter, we analyzed the effect of the single mutations on the expression pattern and G protein activation mediated by the overexpressed N-terminally HiBiT-tagged (expression and activation), and N-terminally HiBiT-tagged and C-terminally Nluc-tagged GPR61 (expression) in HEK293A cells. Our analysis of the cell expression demonstrated that approximately 40% of the total cellularly overexpressed HiBiT-GPR61 WT localizes to the cell membrane. The conclusions are drawn based on the assumption that within the measured bioluminescence counts, which were approximately 100-fold below the detection limit of the plate reader, bioluminescence is linearly proportional to the amount of HiBiT(NanoBiT)-tagged protein present in the wells. Secondly, we also assume that the detergent present in the HiBiT lytic kit does not quench bioluminescence. In a recent publication, the percentage of the cell surface-expressed receptors within the total pool of cellular receptors for an overexpressed HiBiT-GPR61 in CHO cells (with a different GPR61 gene insert without a signal peptide) was lower and equal to ca. 15% (Lees et al, 2023). In our assays, we did not detect statistically significant differences between the mutated and WT receptor variants neither in the bioluminescence elicited from receptors in lysed cells nor in the cell membrane-localized receptors in the experiments with live cells. Therefore we concluded that the identified mutations did not affect membrane trafficking of the receptor in comparison with the WT GPR61. Subsequently, we assessed the impact of the mutations on the receptor activation profile. There is no validated agonist for GPR61 available and therefore we could not assess its ligand-mediated activity but were restricted to quantifying its constitutive activity. GPR61 couples to G_s_ and we employed 2 experimental paradigms to measure G_s_-linked activity of this receptor: EPAC-derived FRET-based biosensor for cAMP and an ebBRET-based G_s_ translocation/activation assay. In the cAMP assay, overexpression of the HiBiT-GPR61 plasmid DNA encoding the mutations T92P^2.56^, R236C^5.66^, and R262C increased the FRET ratio in comparison with the WT which was indicative of the reduction in the GPR61-mediated cAMP accumulation. However, in an orthogonal G_s_ translocation assay, it was only R236C^5.66^ that led to statistically significant differences in ebBRET between itself and the WT receptor. We hypothesize, that the differences between the results from cAMP and G_s_ activation experiments can originate in: (1) supposedly different signal amplifications in the 2 assay paradigms, and (2) a lack of linear correlation between G_s_ activation and cAMP production (Brands et al, 2024; Pizzoni et al, 2024). The constitutive activity of the receptors increased to some extent with the increase in the transfected plasmid amounts and showed statistically significant differences between the WT and R236C^5.66^. Interestingly, we also demonstrated that an overexpressed HiBiT-GPR61 seems unable to signal from intracellular compartments but the introduction of the R236C^5.66^ leads to an increase in ebBRET which reached statistical significance in the Golgi but showed a positive tendency also in the endosomes and the endoplasmic reticulum. These data suggest that this mutant could couple unproductively to G_s_ in these compartments. Next, the data on the G_s_ activation profile in different compartments are supplemented with data on subcellular expression of both receptor variants, overexpressed as C-terminally Nluc-tagged proteins. These constructs show some differences in the subcellular compartment expression, with the WT showing more expression at the cell membrane and the late endosomes—explaining its higher cell surface expression—and R236C^5.66^ expressed at higher levels in the early endosomes and the endoplasmic reticulum. Additionally, we also detected a decrease in basal G_i_ activation upon overexpression of the HiBIT-GPR61 variants. In addition to validating the textbook knowledge of opposing roles of G_s_- and G_i_-coupled receptors, these data can potentially be explained by the effect that overexpressed receptors have on the availability of G proteins and expression levels of endogenous GPCRs (Tubio et al, 2010). Still, this decrease in G_i_ activation was lower for the R236C^5.66^ mutant in comparison with the other variants further proving that this variant reduces basal G_s_-linked activation of the receptor.

Finally, we performed MD experiments to simulate the molecular landscape of the GPR61 carrying C236^5.66^ in comparison to WT. Our MD simulations on the R236C^5.66^ mutant showed that the arginine to cysteine mutation alters the arrangement of helix 5. The residue interaction contacts were different between the 2 conformations, with the WT establishing 5 contacts and the mutant having 3. This difference in contacts in the mutant can be attributed to the unique chemical properties of cysteine. Cysteine’s thiol group (–SH) is more reactive and capable of forming additional interactions, potentially through increased hydrogen bonding or disulfide bridge formation, leading to greater interaction density. The MD simulations also demonstrate a different orientation of the ICL3 between the mutant and the WT receptor which could potentially intercalate G_s_ coupling. However, the ICL3 had to be modeled in our system and therefore these are only weak predictions. Overall, these structural and interaction differences could explain the mutation's impact on protein stability and function despite maintaining similar overall contact patterns. The MD simulations of T92P^2.56^ or R262C mutants did not show striking differences in comparison to the WT.

The obvious limitation of this study is that the lack of a GPR61 agonistic ligand substantially restricts our analyses of mutants’ activity. It also remains unclear if and how results from studies employing overexpressed receptors in HEK293 cells can be translated to native settings to draw any conclusions on signaling and the role of GPR61 in obesity, particularly given that the levels of physiological expression of GPR61 RNA and protein in seemingly the most relevant organ for metabolism and food intake regulation, brain are only moderate (https://www.proteinatlas.org/ENSG00000156097-GPR61). Similarly, the expression of endogenous GPR61 protein in common human cell lines is below detection limits. Nevertheless, we conclude that our findings contribute to the general knowledge about GPR61’s role in health and disease, and add relevant information about severe obesity-linked mutations of this receptor in the overexpressed conditions. Our data lay the basis for further studies on the mechanistic understanding of this receptor and its role in obesity in (patho-)physiologically-relevant models.

## Conflict of interest

No author has an actual or perceived conflict of interest with the contents of this article.
