# Supplementary material for: Identification and molecular characterization of missense mutations in orphan G protein–coupled receptor GPR61 occurring in severe obesity
Source: Mol Pharmacol. 2025 Mar 4;107(4):100026. doi: 10.1016/j.molpha.2025.100026 (PMC12060159; doi:10.1016/j.molpha.2025.100026)
Supplement: Supplementary Material [file mmc13.pdf]

Identification and molecular characterization of missense mutations in orphan GPCR GPR61 occurring in severe obesity

Choi Har Tsang[1], Alexander De Rosa[1,2], Paweł Kozielewicz[1]

[1] Molecular Pharmacology of GPCRs, Department of Physiology and Pharmacology, Karolinska Institutet, 171 65 Solna, Sweden

[2] School of Engineering Sciences (SCI), KTH Royal Institute of Technology, 114 28 Stockholm, Sweden

Corresponding author:
Paweł Kozielewicz, PhD
e-mail: pawel.kozielewicz@ki.se

Supplemental data :

Supplemental Figures 1-9

Supplementary files: final frames from GPR61 WT simulations - WT_1.pdb, WT_2.pdb, WT_3.pdb

GPR61 T92P$^{2.56}$ simulations – T92P_MD1.pdb, T92P_MD2.pdb, T92P_MD3.pdb

GPR61 R236C$^{5.66}$ simulations – R236C_MD1.pdb, R236C_MD2.pdb, R236C_MD3.pdb

GPR61 R262C simulations – R262C_MD1.pdb. R262C_MD2.pdb, R262C_MD3.pdb

A.

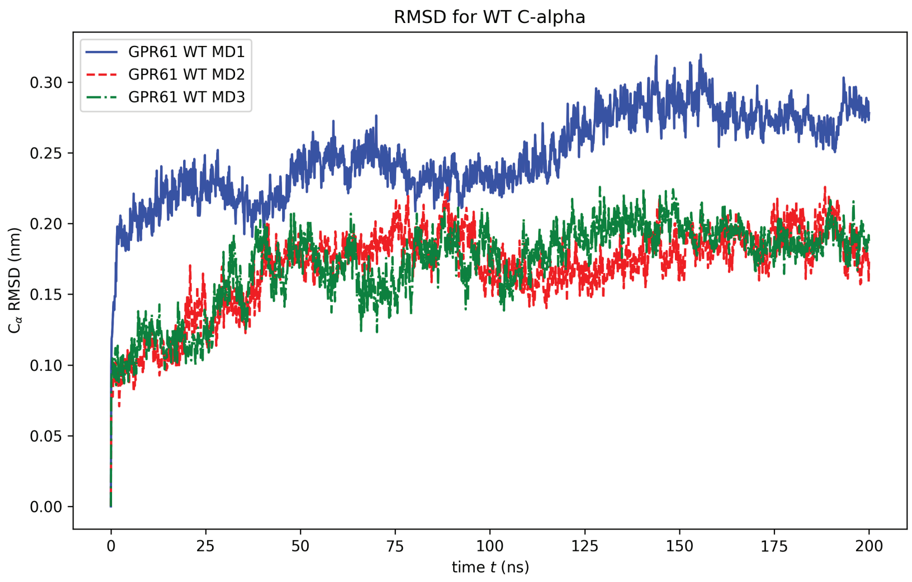

B.

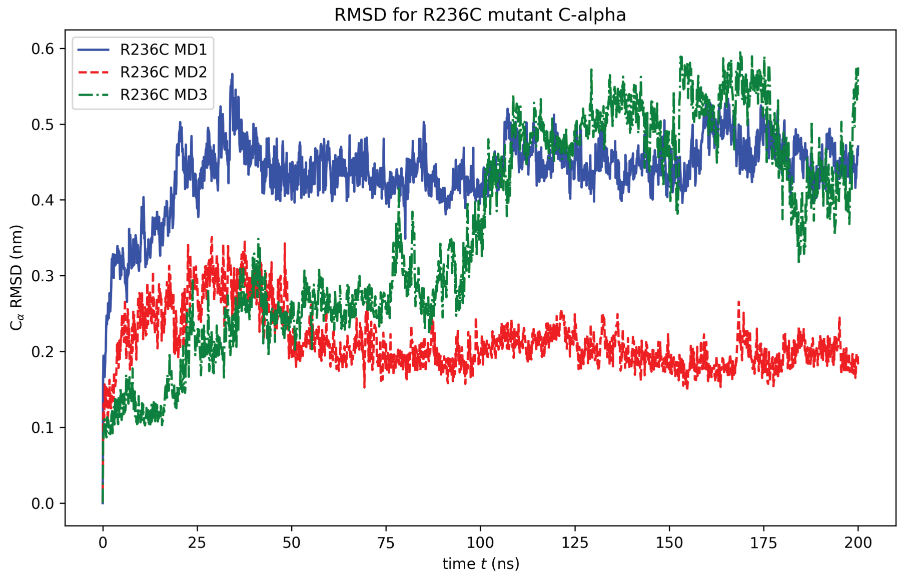

Supplemental Figure 1. RMSD of carbon alpha atoms from the three simulations of the GPR61 WT (**A**) and GPR61 R236C$^{5.66}$ (**B**). Final frames are attached as supplementary material .pdb files: GPR61 WT simulations - WT_1.pdb, WT_2.pdb, WT_3.pdb; GPR61 R236C$^{5.66}$ simulations – R236C_MD1.pdb, R236C_MD2.pdb, R236C_MD3.pdb.

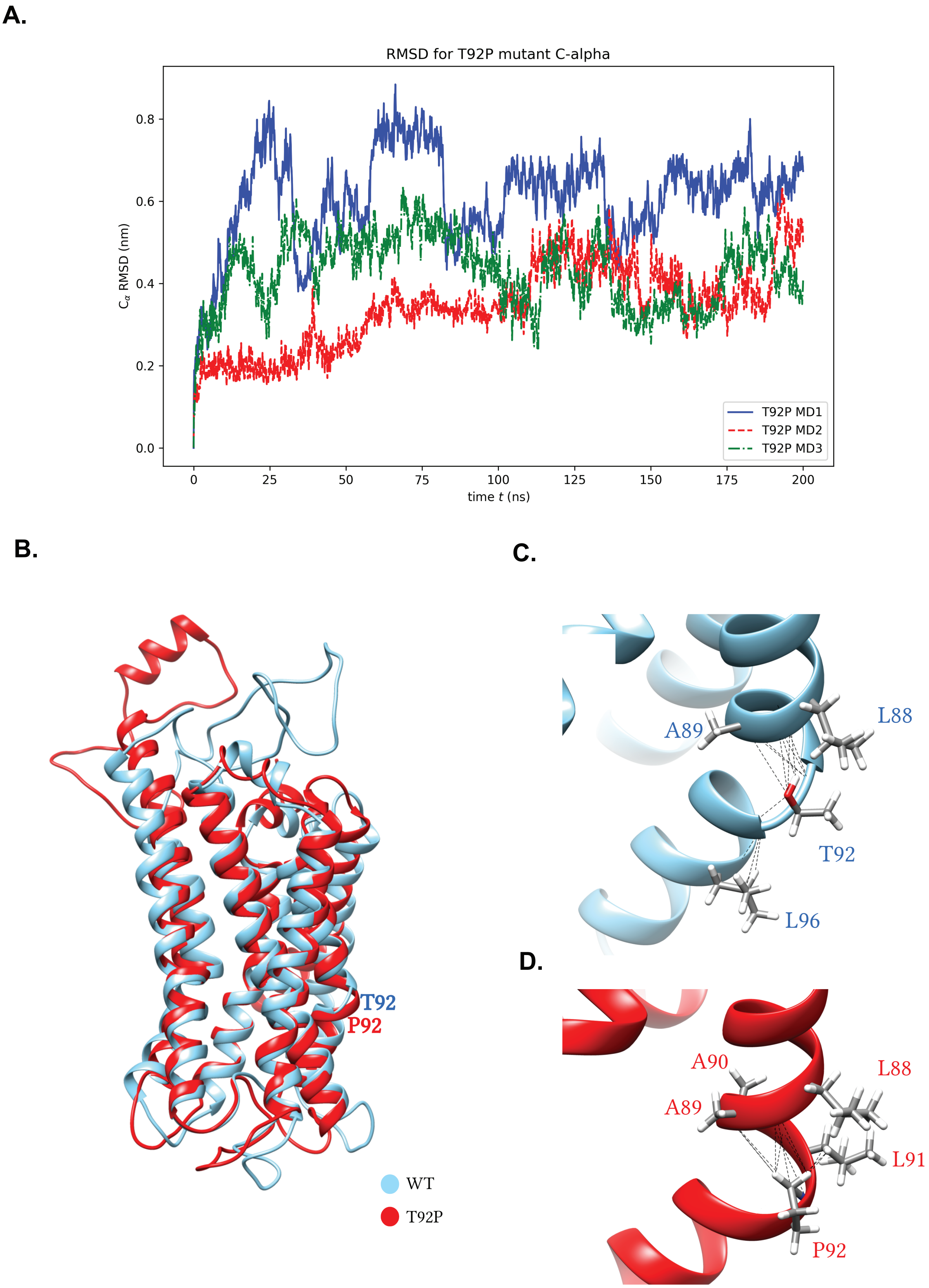

Supplemental Figure 2. A. RMSD of carbon alpha atoms from the three simulations of the GPR61 T92P$^{2.56}$. **B.** Final receptor conformations after 200 ns simulation from one representative MD simulation. **C.** Zoomed-in region of T92$^{2.56}$ (wild-type) and **D.** P92$^{2.56}$ (mutant) with polar interactions depicted as dashed lines. Final frames are attached as supplementary material .pdb files: GPR61 T92P$^{2.56}$ simulations – T92P_MD1.pdb, T92P_MD2.pdb, T92P_MD3.pdb.

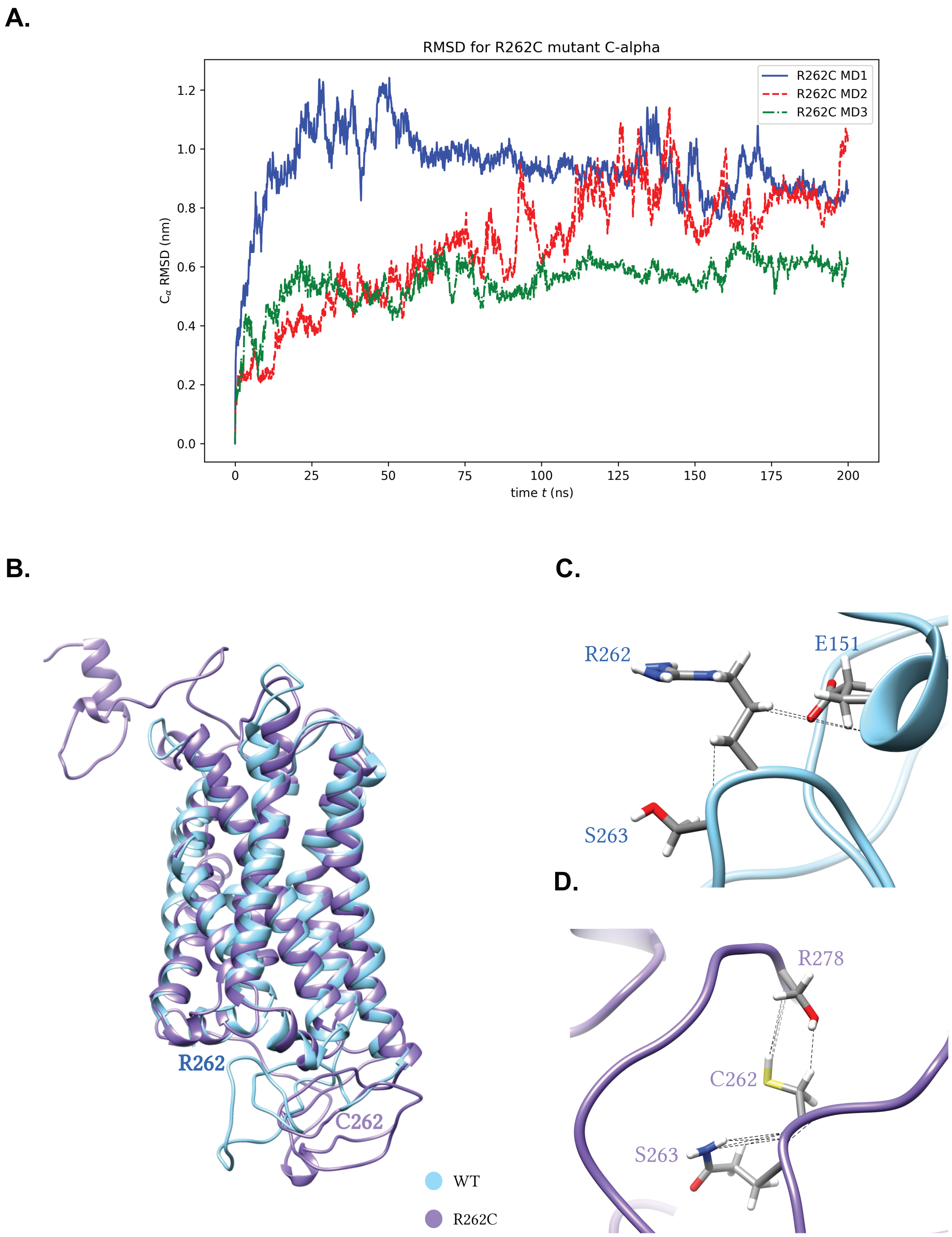

Supplemental Figure 3. A. RMSD of carbon alpha atoms from the three simulations of the GPR61 R262C. **B.** Final receptor conformations after 200 ns simulation from one representative MD simulation. **C.** Zoomed-in region of R262 (wild-type) and **D.** C262 (mutant) with polar interactions depicted as dashed lines. Final frames are attached as supplementary material .pdb files: GPR61 R262C simulations – R262C_MD1.pdb. R262C_MD2.pdb, R262C_MD3.pdb.

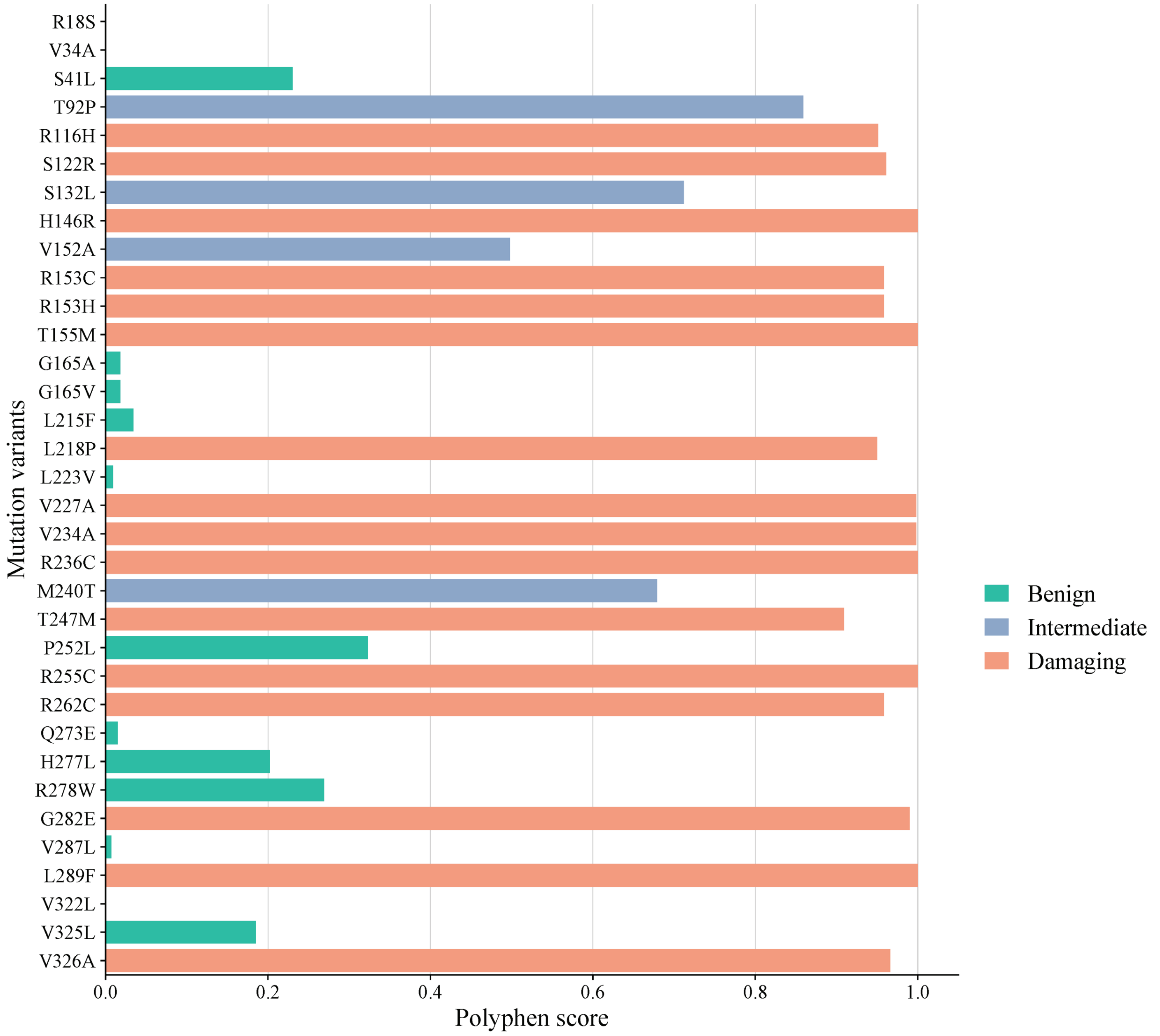

Supplemental Figure 4. PolyPhen-2 analysis of the 34 mutations in GPR61 to predict their impact on structure and function of the protein.

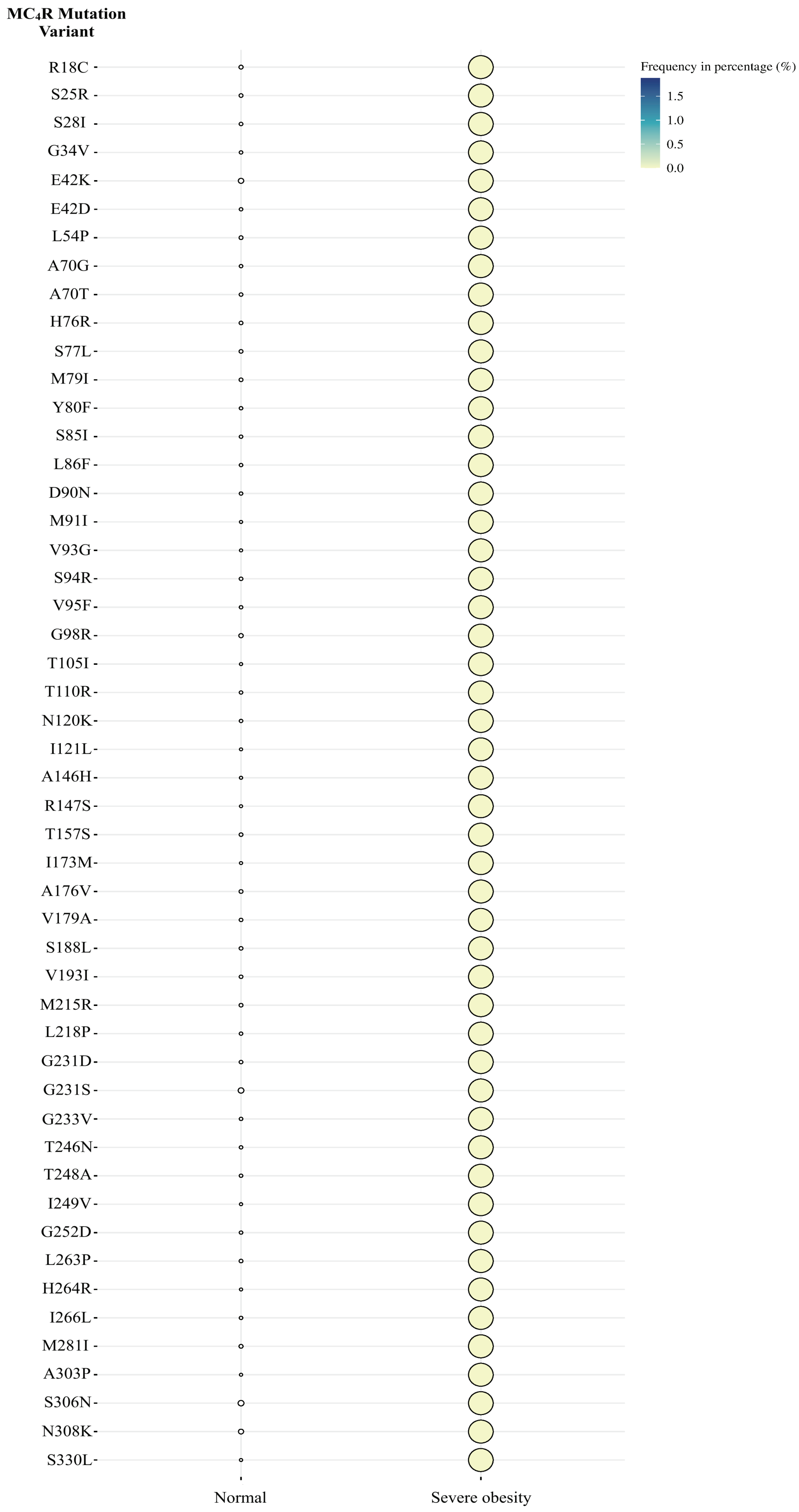

Supplemental Figure 5. Mutational landscape of MC_4R in normal population and then the same mutations in severe obesity patients. Data are presented as frequencies.

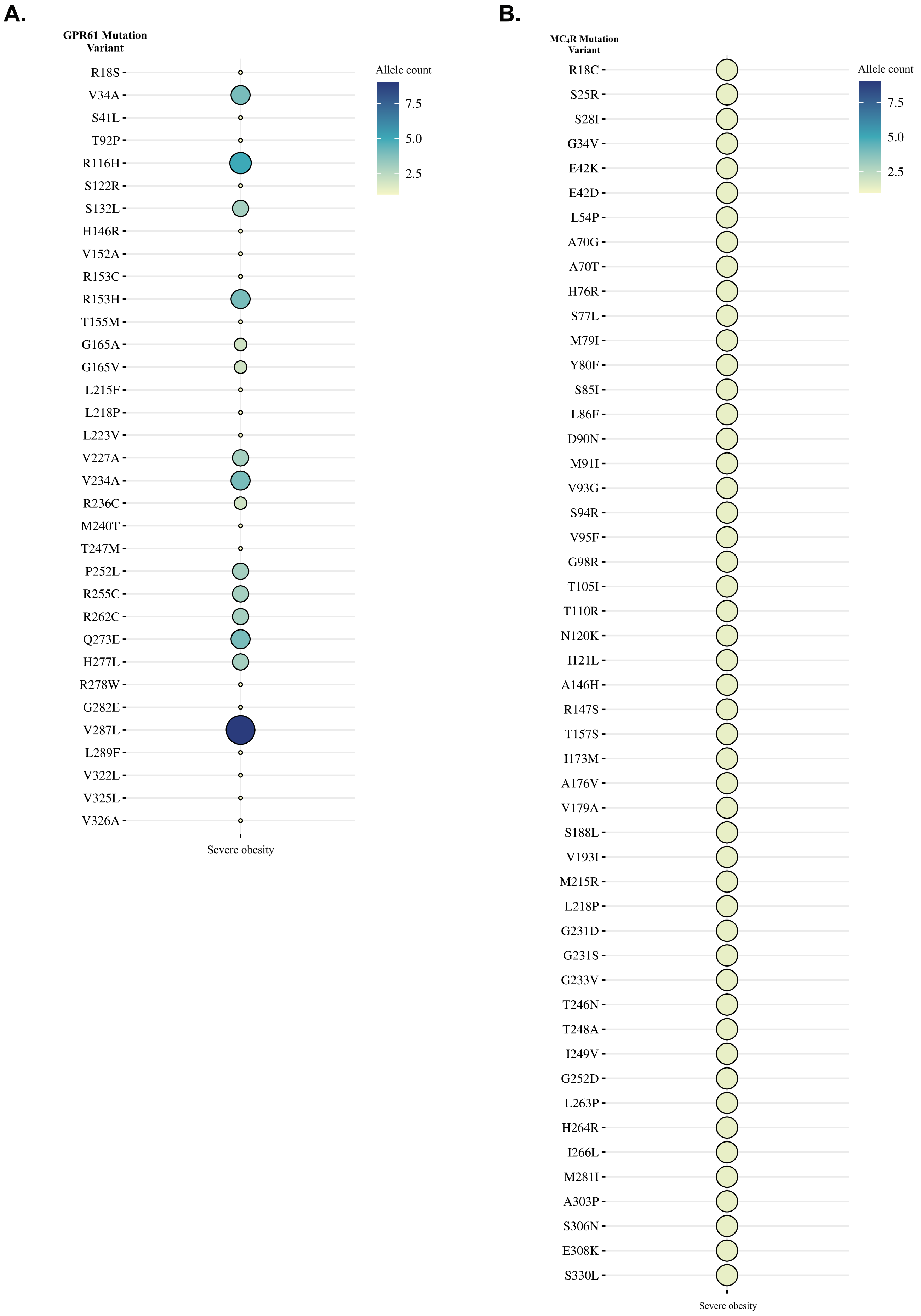

Supplemental Figure 6. Mutational landscape of GPR61 **(A)** and MC4R **(B)** in severe obesity patients. Data are presented as allele counts.

A. **B.**

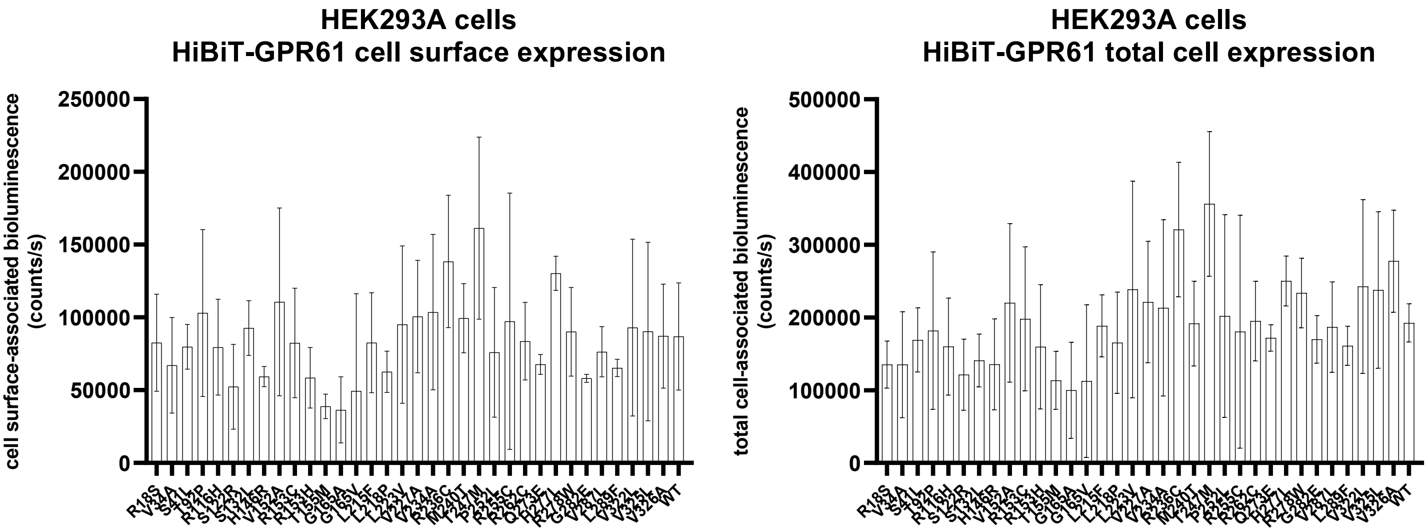

Supplemental Figure 7. A. NanoBiT-emmitted luminescence associated with overexpressed HiBiT-GPR61 variants measured in live cells (cell surface expression). **B.** NanoBiT-emmitted bioluminescence associated with overexpressed HiBiT-GPR61 variants following cell lysis (total cell expression); mean +/- s.d. of n=3 independent experiments.

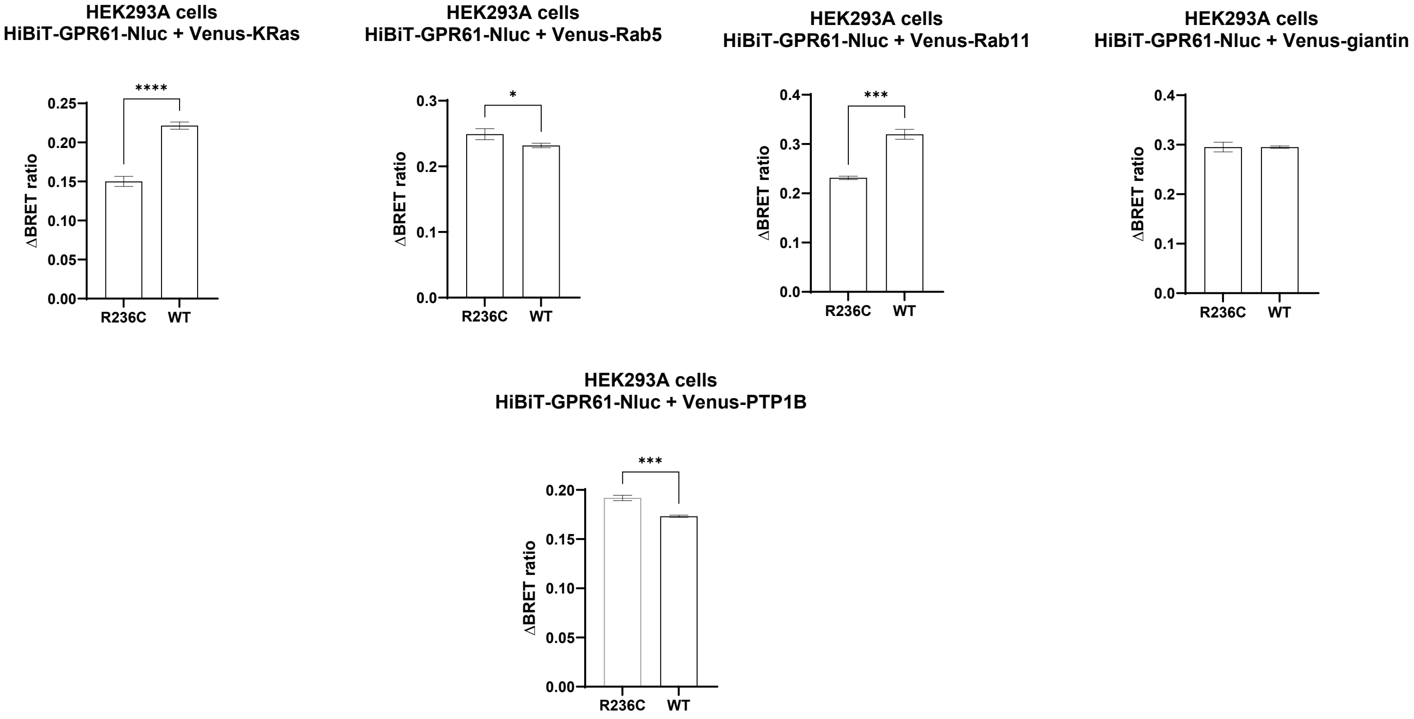

Supplemental Figure 8. Bystander BRET assay to measure presence of overexpressed HiBiT-GPR61-Nluc WT and R236C$^{5.66}$ at cellular compartments. The assay showed that the WT construct has a higher expression at the cell surface and in the late/recycling endosomes, while the mutant is expressed to a higher degree in the early endosomes and the endoplasmic reticulum. Statistical analysis was performed with *t*-test; significance levels are given as: *P < 0.05; **P < 0.01; ***P < 0.001; ****P < 0.0001; mean +/- s.d. of n= 3 independent experiments.

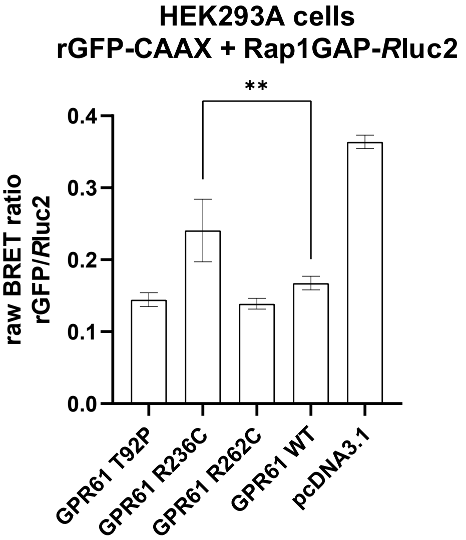

Supplemental Figure 9. ebBRET assay to measure Gi protein activation. The presence of overexpressed GPR61 R236C$^{5.66}$ leads to a lower, statistically-significant in comparison to the WT, T92P$^{2.56}$ and R262C, reduction in the bystander BRET ratio between Rap1Gap-*R*luc2 and rGFP-CAAX. These data indicate that overexpression of HiBiT-GPR61 leads to a reduction in basal Gi activation in HEK293A cells. Data were analyzed for differences between the mutants with the WT by one-way ANOVA with Dunnett's post-hoc analysis. Significance levels are given as: *P < 0.05; **P < 0.01; ***P < 0.001; ****P < 0.0001; mean +/- s.d. of n=3 independent experiments.
